# Carotid Atherosclerotic Disease Predicts Cardiovascular Events in Hemodialysis Patients: A Prospective Study

**DOI:** 10.1371/journal.pone.0127344

**Published:** 2015-06-01

**Authors:** Sílvia Collado, Elisabeth Coll, Carlos Nicolau, Mercedes Pons, Josep M Cruzado, Julio Pascual, Aleix Cases

**Affiliations:** 1 Nephrology Department, Hospital del Mar, Barcelona, Spain; 2 Nephrology Department, Fundació Puigvert, Barcelona, Spain; 3 Radiology Department, Hospital Clínic, Barcelona, Spain; 4 CETIRSA Barcelona, Fresenius Medical Care, Barcelona, Spain; 5 Institut Hemodiàlisi Barcelona, Diaverum, Barcelona, Spain; 6 Nephrology Department, Hospital Clínic and Universitat de Barcelona, Barcelona, Spain; Temple University School of Medicine, UNITED STATES

## Abstract

**Background:**

To evaluate the predictive value of carotid atherosclerotic disease (CAD) and intima-media thickness (IMT) on incident cardiovascular disease and mortality in hemodialysis patients.

**Methods:**

Multicenter, observational, prospective study including 110 patients, followed-up to 6 years. Carotid doppler ultrasonographic findings were classified in 4 degrees of severity: 1) IMT <0.9 mm, 2) IMT >0.9 mm, 3) carotid plaque with stenosis <50% and 4) plaque with stenosis >50%. The associations between IMT and CAD and cardiovascular events, total and cardiovascular mortality were assessed.

**Results:**

83% of the patients had atherosclerotic plaques (CAD degrees 3-4). During follow-up, 29.1% of patients experienced cardiovascular events, and 28.2% died, 38.7% of cardiovascular origin. The presence of plaques was associated with cardiovascular events (p = 0.03) while calcified plaques were associated with both cardiovascular events (p = 0.01), cardiovascular mortality (p = 0.03) and non-significantly with overall mortality (p = 0.08) in the survival analysis. Carotid IMT was not associated with outcomes. Cardiovascular events correlated with CAD severity (HR 2.27, 95% CI 1.13-4.54), age (HR 1.04, 1.01-1.06), previous cardiovascular disease (HR 1.75, 1.05-4.42), dyslipidemia (HR 2.25, 1.11-4.53), lipoprotein (a) (HR 1.01, 1.00-1.02), troponin I (HR 3.89, 1.07-14.18), fibrinogen levels (HR 1.38, 0.98-1.94) and antiplatelet therapy (HR 2.14, 1.04-4.4). In an age-adjusted multivariate model, cardiovascular events were independently associated with previous coronary artery disease (HR 3.29, 1.52-7.15) and lipoprotein (a) (HR 1.01, 1.00-1.02).

**Conclusions:**

The presence of carotid plaques and, especially, calcified plaques, are predictors of new cardiovascular events and cardiovascular mortality in hemodialysis patients, while IMT was not. The prognostic value of calcified plaques should be confirmed in future studies.

## Introduction

Cardiovascular (CV) disease is the main cause of mortality in hemodialysis (HD) patients [1–4]. However, the high prevalence of CV disease and traditional CV risk factors, such as hypertension, diabetes, smoking, dyslipidemia, sedentary lifestyle, or left ventricular hypertrophy, do not fully explain this increased CV risk in uremic patients [5,6]. In fact, the Framingham Risk Score underestimates the CV risk in HD patients, thus suggesting that other factors are involved in the development and progression of the CV disease in these patients [5]. In recent years, several markers have been reported to have prognostic implications in dialysis patients [7]. Among them, an increased carotid intima-media thickness (IMT) has been associated with CV disease and risk of stroke, both in the general population and in HD patients [8,9]. This parameter is associated with traditional and emerging CV risk factors, as well as with the progression, stabilization, and regression of atherosclerosis with lipid-lowering and antihypertensive treatments in the general population [10–13].

On the other hand, the presence of carotid plaques is considered as target organ damage by the ESH-ESC Practice Guidelines for the Management of Hypertension, and it is an independent marker of increased risk and the likelihood of CV events or mortality in HD patients [9,14,15]. It is tempting to speculate that, while IMT may be a better marker of arteriosclerosis and arterial stiffness, which are highly prevalent in HD patients, carotid atherosclerosis (presence of plaques) is a marker of subclinical atherosclerosis and a better predictor of CV events than IMT in these patients.

Currently, the assessment of IMT and carotid plaques is not uniform, making it difficult to interpret and compare the results between studies [16]. In addition, there are only few long–term prospective studies evaluating the prognostic value of carotid ultrasound findings in the HD population [9,15,17,18].

The aim of this study was to assess the predictive value of carotid IMT, the presence of carotid atherosclerotic plaques and their characteristics, on the incidence of CV disease and overall and CV mortality in HD patients.

## Material and Methods

### Design

This was a multicenter, observational, cross-sectional and prospective study including 110 adult patients with end-stage renal disease on maintenance HD for at least 6 months from the Hospital Clinic and 4 satellite HD units. Patients had to be clinically stable and without evidence of clinical heart failure at the time of enrollment. Clinical heart failure was defined as dyspnea plus two of the following conditions: increased jugular venous pressure, bibasilar pulmonary rales, pulmonary venous hypertension or interstitial edema requiring hospitalization or ultrafiltration and/or a left ventricular ejection fraction <25%. Patients agreed to participate in the study and signed an informed written consent. The study was approved by the Ethics’ Committee of the Hospital Clinic (Barcelona, Spain).

All patients underwent a carotid Doppler ultrasound study, evaluating the common carotid, bifurcation and the origin of the internal carotid artery, analyzing the presence of plaques, calcifications and degree of stenosis (1–4) [16], in addition to measuring the carotid IMT according to the Mannheim Consensus 2006. Carotid atherosclerotic disease (CAD) was classified in 4 degrees of severity (Grade 1: IMT <0.9 mm, grade 2: IMT > 0.9 mm, grade 3: presence of carotid plaques with stenosis <50% and grade 4: presence of carotid plaques with stenosis >50%).

At baseline, anthropometric, demographic, clinical and analytical data were collected at baseline: age, sex, time on HD, previous kidney transplants, and etiology of renal disease; history of CV risk factors (hypertension, diabetes, hypercholesterolemia, smoking); characteristics of the HD, and concomitant treatments (antihypertensive, lipid-lowering and antiplatelet/anticoagulant agents, vitamin D analogs, erythropoiesis stimulating agents and doses of calcium and intravenous iron). The Charlson comorbidity index was also calculated. Patients were followed up for a maximum of 6 years, and events including: CV disease of cardiac origin (defined as coronary artery disease events, congestive heart failure or cardiac arrhythmias), cerebrovascular events (stroke or transient ischemic attack) and peripheral arterial events (peripheral artery disease, mesenteric ischemia, etc.), as well as total and CV mortality were collected, until death, loss to follow-up or kidney transplantation. The associations of CAD and IMT with CV events, CV and overall mortality were prospectively assessed.

### Methods

Blood pressure was measured before each HD session during one week. Blood samples were obtained before the second dialysis session of the week after 20–30 minutes of rest in a supine position. At baseline, the following biochemical and hematological parameters were also measured: calcium, phosphorus, CaxP product, iPTH, total cholesterol, LDL-cholesterol, triglycerides, hemoglobin, fibrinogen, ferritin, C reactive protein (CRP), blood urea nitrogen (BUN) and Kt/V. Additional special tests included: lipoprotein (a), homocysteine, troponin-I and brain natriuretic peptide (BNP).

Carotid Doppler ultrasound was performed with the model color Doppler ultrasound equipment and software to measure IMT. We used a high resolution linear transducer with 7.5 MHz of frequency and 0.1 mm of resolution in real time image and 3.75 MHz for Doppler. Subjects were placed in a supine position with the head rotated 45°, contralateral to the examined carotid. Three segments were examined: the common carotid artery at 1 cm proximal to the carotid bulb, the carotid bifurcation (1–2 cm) and the origin of the internal carotid (1 cm distal to the bifurcation) bilaterally. Carotid IMT was measured at 1 cm prebifurcation, explored in a longitudinal section on the far wall, obtaining 4 measurements at regular intervals. We calculated the average of 8 measurements, right and left, considering as a normal value an IMT ≤ 0.9 mm, according to the criteria of the European Guidelines on Hypertension of the ESC-ESH of 2007 [14]. We defined the presence of plaque as a focal structure that invaded the arterial lumen of at least 0.5 mm or >50% of the surrounding IMT or demonstrated a thickening >1.5 mm measured from the media-adventitia interface to the intima-lumen interface [16]. For plaque evaluation, longitudinal and transverse section in B mode were performed and then, analyzed in each vessel by color Doppler and describing the location, number and structural ultrasonographic features, including calcification.

### Statistical analysis

Data were analyzed using the software Statistical Package for the Social Sciences (SPSS, version 20.0, SPSS Inc). Comparison of continuous variables was performed by using the Student's t test for unpaired data and the Chi-square test for qualitative variables. If continuous variables did not show a normal distribution, data were analyzed using the Mann-Whitney´s U test. Survival analysis was carried out by using the Kaplan-Meier test and multivariate Cox regression analysis. A P-value <0.05 was considered significant.

## Results

### Demographics and baseline assessment

The demographic parameters of the 110 patients included are summarized in Table 1. Sixty nine percent were male, mean age was 58.9 ± 15.3 years and median time on HD 37 [14.75, 85.5] months. The mean Charlson comorbidity index was 5.26 ± 2.18 points, and 27.3% of the patients had had a previous renal transplant. The most common causes of end-stage renal disease were vascular (19.1%) and glomerular (19.1%), followed by unknown etiology (16.4%) and diabetic nephropathy (15.5%). The prevalence of CV disease at baseline was 52.7%: 46.4% of patients had a history of cardiac disease and 17.3% had non-cardiac vascular disease (cerebrovascular disease or peripheral artery disease). The prevalence of traditional CV risk factors is included in Table 1.

**Table 1:** Descriptive analysis of the hemodialysis population and carotid ultrasound findings.

	(Mean ± SD)(Cases/%)
**Patients (n)**	110
**Age (years)**	58.9 **±** 15.3
**Sex (male/female)**	76 (69.1%)/34 (30.9%)
**Charlson Comorbidity Index**	5.26 **±** 2.18
**Body mass index (Kg/m** ^**2**^ **)**	24.33 **±** 4.39
**Time on HD (months)**	37 [14.75, 85.5]
**Dialysis dose (eKt/V)**	1.35 **±** 0.22
**Previous kidney transplant**	30 (27.3%)
**Previous heart disease**	51 (46.4%)
**Previous cerebrovascular or peripheral artery disease**	19 (17.3%)
**Mean blood pressure (mmHg)**	90.72 **±** 13.18
**Tobacco use (yes/no/former)**	23/63/24 (20.9%/57.3%/21.8%)
**Diabetes mellitus (yes/no)**	24 (21.8%)/86 (78.2%)
**Hypertension (yes/no)**	94 (85.5%)/16 (14.5%)
**Dyslipidemia (yes/no)**	43 (39.1%)/67 (60.9%)
**Left ventricular hypertrophy (yes/no)**	34 (30.9%)/76 (69.1%)
**IMT mean (mm)**	0.78 **±** 0.28 mm
**IMT > 0.9 mm**	37 (40.2%)
**Presence of Plaques**	93 (83.6%)
**Calcified plaques**	80 (72.7%)
**Presence of Stenosis**	43 (39.1%)
**< 50%**	32 (29.1%)
**50–70%**	8 (7.3%)
**> 70%**	2 (1.8%)
**Occlusion**	1 (0.9%)
**Stage classification according to IMT and plaques**	
**Grade 1 (IMT <0.9 mm, no plaques)**	17 (15.5%)
**Grade 2 (IMT >0,9 mm, no plaques)**	1 (0.9%)
**Grade 3 (Plaque with stenosis <50%)**	81 (73.6%)
**Grade 4 (Plaque with stenosis >50%)**	11 (10%)

HD: Hemodialysis. IMT: Intima media thickness.

Mean IMT was 0.78 **±** 0.28 mm (n = 92). In 40.2% of the cases, IMT was abnormal. The presence of carotid plaques was very common (83.6% of patients), showing some degree of stenosis in 43 of them (39.1%). Furthermore, most patients had their plaques calcified (72%). Thus, most of our patients were allocated to the more severe degrees of our CAD classification (degrees 3–4) (Table 1 and S1 Fig).

In the univariate analysis, pathological carotid IMT was positively associated with age, male sex, previous CV disease, smoking, Charlson comorbidity index and pulse pressure. And negatively with history of a previous transplant, serum phosphate levels and calcium-phosphorus product (Table 2).

**Table 2:** Association between abnormal intima-media thickness (IMT) and plaques with clinical and laboratory parameters.

	IMT >0.9 mm (n = 37)	IMT <0.9 mm (n = 55)	P-Value (IMT>0,9 vs <0,9)	Plaques(n = 92)	No plaques(n = 18)	P-Value (plaques vs no plaques)
**Sex (M/F)**	31/6	32/23	**0.01**	64/28	12/6	0.78
**Age (years)**	66.3 **±** 12.7	52.7 **±** 14.1	**<0.001**	61.7 **±** 14.3	44.7 **±** 12.3	**<0.001**
**Time on HD (months)**	38 [15, 86]	57 [20,144]	0.33	37 [17,84]	39 [11,174]	0.87
**Charlson comorbidity index**	6.16 **±** 2.15	4.4 **±** 1.89	**<0.001**	5.5 **±** 2.17	4.06 **±** 1.86	**0.01**
**Previous kidney transplant (yes/no)**	6/31	21/34	**0.03**	24/68	6/12	0.56
**Tobacco (yes/no)**	21/16	16/39	**0.01**	42/50	5/13	0.19
**Hypertension (yes/no)**	33/4	46/9	0.55	84/8	10/8	**0.001**
**Calcium (mg/dl)**	9.31 **±** 0.85	9.05 **±** 1.0	0.19	9.28 **±** 0.94	8.8 **±** 0.77	0.07
**Phosphate (mg/dl)**	5.09 **±** 1.28	6.04 **±** 1.76	**0.006**	5.71 **±** 1.72	5.76 **±** 1.82	0.9
**Calcium Phosphorus product**	47.2 **±** 11.9	54.4 **±** 15.7	**0.01**	52.7 **±** 15.7	51.3 **±** 17.8	0.73
**Pulse pressure (mmHg)**	63.8 **±** 18	54.6 **±** 17.1	**0.02**	59.9 **±** 16.7	50.0 **±** 14.4	**0.03**
**Arrhythmia (yes/no)**	13/24	5/50	**0.003**	22/70	0/18	**0.02**
**Prevalent cardiovascular disease (yes/no)**	13/24	35/20	**0.01**	41/51	12/6	0.12
**Cardiovascular events (yes/no)**	12/25	12/43	0.33	31/61	1/17	**0.02**
**Intima media thickness (mm)**				0.81 **±** 0.28	0.58 **±** 0.14	**0.04**
**Left ventricular mass (g)**	252.5 **±** 57.8	273.1 **±** 56.4	0.12	273.5 **±** 64.9	228.7 **±** 49.1	**0.01**
**CRP (mg/dl)**	1.05 **±** 1.31	1.08 **±** 0.95	0.9	1.17 **±** 1.2	0.56 **±** 0.59	**0.003**
**Fibrinogen (g/l)**	4.42 **±** 0.97	4.65 **±** 0.81	0.25	4.73 **±** 0.88	3.8 **±** 0.78	**<0.001**

HD: Hemodialysis. CRP: C reactive protein

The presence of atherosclerotic plaques showed a positive correlation with age, hypertension, history of arrhythmia, IMT, Charlson comorbidity index, pulse pressure, left ventricular mass index, CRP and fibrinogen levels. We did not find differences with daily calcium dose or calcium/phosphorous product between patients with calcified plaques and non-calcified plaques.

### Prospective observational study

During a mean follow-up of 3.17 ± 2.07 years, 42 (38.2%) patients received a kidney transplant and 11 patients (10%) were lost to follow-up. Thirty-two patients (29.1%) suffered new cardiac (69%) or vascular events (31%), the most frequent type being coronary artery disease-related events, such as acute myocardial infarction (22%); arrhythmia (19%), heart failure (16%) and peripheral vascular disease (16%). Total mortality was 28.2%, being of CV origin in 38.7% of cases. Among the CV causes of death the most frequent were also myocardial infarction (13%), sudden death (10%) and vascular pathology (9%). Among the non-CV causes of mortality, the most frequent were infectious diseases (26%), followed by bleeding (16%), and cancer (10%) (S1 Table). Patients with previous cardiovascular disease had more new cardiovascular events (36.2%, 21/58) and a higher total mortality (32.75%, 19/58) during the follow-up compared with patients without prevalent cardiovascular disease at baseline (21.15%, 11/52 and 23% 12/52, respectively).

**Fig 1:**
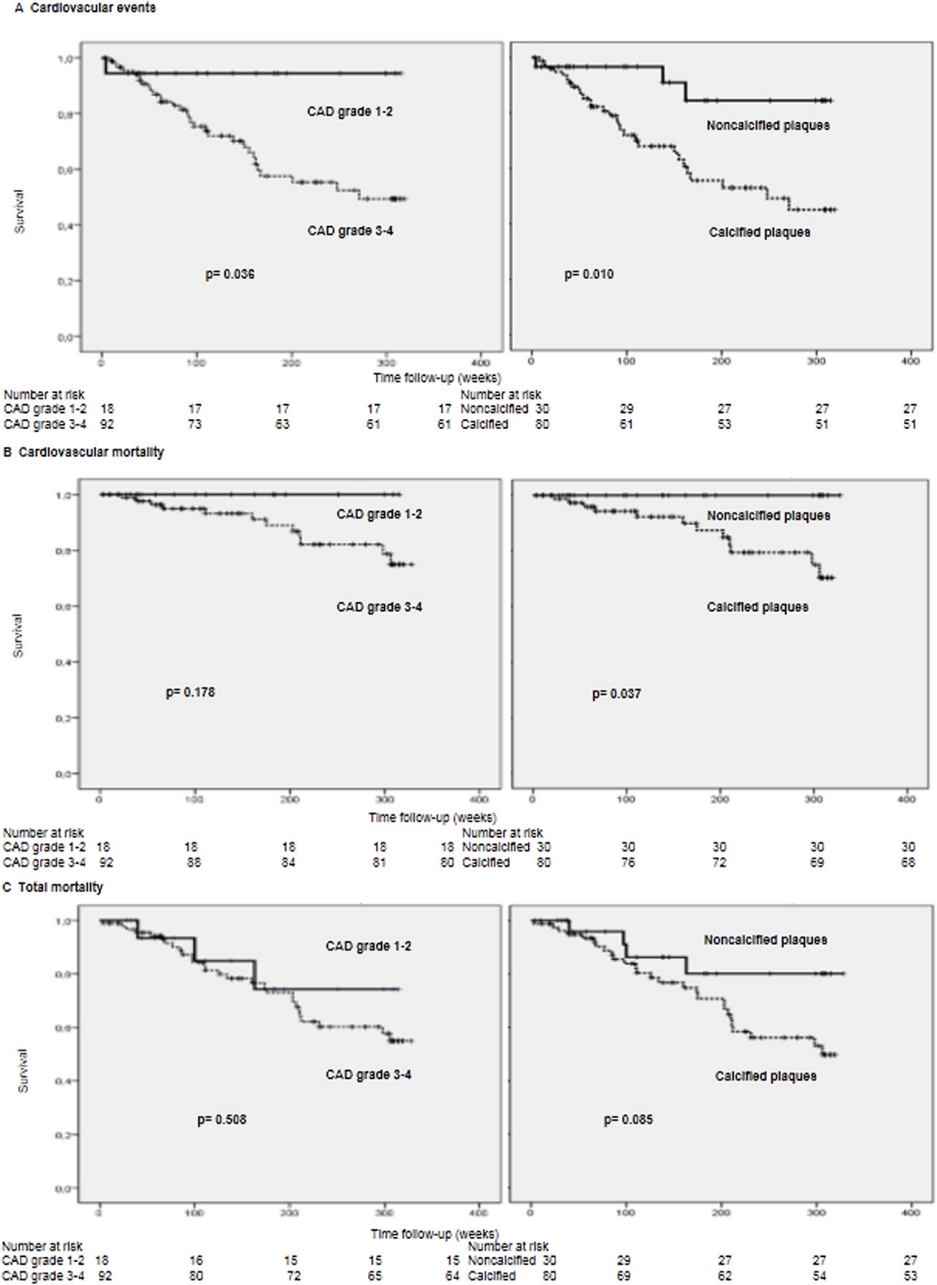
Actuarial survival in patients according to carotid atherosclerotic disease grades (grade 1–2 versus grade 3–4) and calcified versus non-calcified plaques. A) Survival analysis of patients free of cardiovascular events. B) Cardiovascular mortality-free patients. C) Overall mortality free patients.

In the Kaplan Meier survival analysis, the most severe degrees of carotid atherosclerotic disease (degrees 3 and 4) were associated with new CV events (p = 0.03) (Fig 1). Overall and CV mortality were also higher in the group with severe CAD, but without reaching statistical significance.

The presence of calcified plaques was associated with new CV events (p = 0.01), CV mortality (p = 0.03) and non-significantly with overall mortality in the survival analysis (p = 0.08) (Fig 1).

Carotid IMT did not correlate with new CV events, overall or CV mortality in our analysis.

### Cox regression analysis and multivariate analysis

In the univariate Cox regression analysis, new cardiovascular events showed a positive correlation with CAD severity (HR 2.27, 95% CI 1.13–4.54, p = 0.02), age (HR 1.04, 95% CI 1.01–1.06, p = 0.004), previous CV disease (HR 1.75, 95% CI 1.05–4.42, p = 0.03) and, especially, coronary artery disease (HR 4.14, 95% CI 2.05–8.37, p<0.001), as well as, dyslipidemia (HR 2.25, 95% CI 1.11–4.53, p = 0.02), lipoprotein (a) (HR 1.01, 95% CI 1.00–1.02, p = 0.02), troponin I (HR 3.89, 95% CI 1.07–14.18, p = 0.03), antiplatelet therapy (HR 2.14, 95% CI 1.04–4.4, p = 0.03) and fibrinogen levels (HR 1.38, 95% CI 0.98–1.94, p = 0.05) (Table 3). And negatively with diastolic blood pressure (HR 0.97, 95% CI 0.95–1.00, p = 0.05). All these significant values were included in a stepwise multivariate Cox model. In the final multivariate model, new CV events were independently associated with age (HR: 1.04, 95% CI 1.01–1.06, p = 0.007), history of coronary artery disease (HR: 3.29, 95% CI 1.52–7.15, (p = 0.003) and lipoprotein (a) (HR: 1.01, 95% CI 1.00–1.02, p = 0.02) (Table 3).

**Table 3:** Univariate and Multivariate Cox regression analyses for cardiovascular events in hemodialysis patients.

	Univariate analysis		Multivariate analysis	
**Variable**	**HR (CI 95%)**	**P-Value**	**HR (CI 95%)**	**P-Value**
**Age (years)**	1.04 (1.01–1.06)	**0.004**	1.04 (1.01–1.06)	**0.007**
**CAD (yes/no)**	2.27 (1.13–4.54)	**0.02**		
**Previous cardiovascular disease (yes/no)**	1.75 (1.05–4.42)	**0.03**		
**Previous coronary heart disease (yes/no)**	4.14 (2.05–8.37)	**<0.001**	3.29 (1.52–7.15)	**0.003**
**Dyslipidemia (yes/no)**	2.25 (1.11–4.53)	**0.02**		
**Diabetes Mellitus (yes/no)**	0.91 (0.39–2.12)	0.84		
**Hypertension (yes/no)**	2.11 (0.64–6.95)	0.22		
**Tobacco (yes/no)**	1.19 (0.59–2.38)	0.62		
**SBP (mmHg)**	0.99 (0.98–1.01)	0.54		
**DBP (mmHg)**	0.97 (0.95–1.00)	**0.05**		
**PP (mmHg)**	1.00 (0.98–1.02)	0.62		
**Total cholesterol (mg/dl)**	0.99 (0.98–1.01)	0.54		
**LDL-c (mg/dl)**	1.00 (0.98–1.01)	0.87		
**Lipoprotein (a) (mg/dl)**	1.01 (1.00–1.02)	**0.02**	1.01 (1.00–1.02)	**0.02**
**CRP (mg/dl)**	1.21 (0.95–1.54)	0.11		
**Troponin I (ng/ml)**	3.89 (1.07–14.18)	**0.03**		
**Fibrinogen (g/l)**	1.38 (0.98–1.94)	**0.05**		
**Antiplatelet therapy (yes/no)**	2.14 (1.04–4.40)	**0.03**		
**ACE inhibitors / ARB treatment (yes/no)**	0.15 (0.02–1.11)	0.06		

CAD: carotid atherosclerosis disease, SBP: systolic blood pressure, DBP: diastolic blood pressure, PP: pulse pressure, CRP: C reactive protein, ACE: angiotensin converting enzyme, ARB: Angiotensin receptor blockers

## Discussion

The main results of our study are the high prevalence of carotid atherosclerosis and the predominance of calcified plaques in hemodialysis patients. The presence of carotid plaques, rather than IMT, predicted new cardiovascular events during the follow-up. Furthermore, the presence of calcified plaques predicted both new cardiovascular events, as well as CV mortality, thus suggesting the additional predictive value of plaque calcification in hemodialysis patients.

The presence of carotid plaques has been reported to be present in more than 60% of hemodialysis patients in several studies [9,19–21]. An increased plaque burden and a higher prevalence of plaque calcification has been reported in this population vs the general population [21,22]. In hemodialysis patients, authors have found association with different types of carotid atherosclerosis and CV risk: such as the sum of the thickness of all plaques found [21], with the sum of bilateral location of all plaques [22], while others propose scores based on the number or calcification of plaques [19].

Ultrasonographic and necropsic studies have shown a much higher prevalence of calcified plaques in ESRD patients than in age-matched controls, in whom soft plaques are more frequent [20,23]. Although it has been suggested that calcified plaques are more stable; this concept has been recently challenged in ESRD by some studies that have shown that the atherosclerotic plaques of CKD patients are more complex than those of individuals with normal renal function. Coronary plaques in patients with CKD had a larger lipid core with a higher content of calcium, cholesterol crystals and plaque disruption, as well as intraplaque hemorrhage and new intimal vessels, compared with non-CKD patients [24,25], suggesting that atherosclerotic plaques in CKD patients are more vulnerable, despite the higher presence of calcium.

ESRD is associated with an increased IMT, as well as arteriosclerosis, vascular calcification, and subsequent arterial stiffness [9,26–28]. Although carotid IMT has been traditionally considered a marker of subclinical atherosclerosis in the general population [29] and in dialysis patients [30], recent studies indicate that the addition of this parameter to traditional cardiovascular risk factor prediction models does not significantly improve their performance in the general population [10]. Carotid IMT has been found to be associated with the presence of prevalent CV disease, traditional cardiovascular risk factors (age, male gender, smoking, diabetes, dyslipidemia, hypertension), as well as malnutrition-inflammation status and different biomarkers in hemodialysis patients [9,26,28]. In our study, IMT was associated with age, male sex, prevalent CV disease, smoking, but not with other cardiovascular risk factors or markers of malnutrition-inflammation. Carotid IMT was also associated with pulse pressure, which can be considered a surrogate of arterial stiffness; but did not predict new cardiovascular events or mortality in our patients. Although some studies have shown a relationship between IMT and mortality in hemodialysis [9,31], this relationship may be indirect, since IMT was associated with prevalent cardiovascular disease and the presence of plaques. Thus, in our hands, IMT seems to be a marker of arteriosclerosis, rather than atherosclerosis and CV risk in these patients.

The association of carotid atherosclerosis with cardiovascular events and/or mortality has been previously reported in dialysis patients [19,32]. Benedetto et al [15] further found that the rate of formation of new plaques was a strong, independent predictor of incident cardiovascular events in ESRD, while changes in IMT did not predict cardiovascular outcomes. Several studies in dialysis patients [9,19] and in the general population [10,33] have shown that the presence of plaques is a better prognostic marker than IMT. Our results are in agreement with these previous studies, further supporting that the presence of carotid atherosclerosis is a better predictor of future CV events than IMT.

Furthermore, in our study the presence of calcified plaques was also associated with both fatal and non-fatal cardiovascular events. Our results show the prognostic value of calcified plaques as a cardiovascular risk marker, similarly to previous studies [19,32], and its association with cardiovascular morbidity and mortality in dialysis patients. However, the additional predictive value of plaque composition on the cardiovascular risk in this population deserves further research.

Carotid plaque calcification has been associated to secondary hyperparathyroidism [34], hypoalbuminaemia and the chronic inflammatory state, although other studies failed to find these relationships, neither with age nor with time on dialysis [20]. In our study, in agreement with Savage et al [20] we failed to find a relationship between the presence of calcified plaques with markers of bone mineral metabolism, such as levels of phosphorus, calcium or iPTH; prescribed daily dose of calcium or vitamin D administration, although this issue remains controversial [34].

Lipid abnormalities and serum lipoprotein (a) levels are associated with IMT, the presence and number plaques, suggesting a role for lipoprotein (a) as an independent risk factor for atherosclerosis in this population [35,17]. We found no relationship between lipid abnormalities or Lp (a) and IMT, in contrast to other studies (22), Lp (a) levels were associated with the severity of CAD in the univariate analysis, and with the incidence of cardiovascular events in the multivariate analysis, in agreement with previous studies [36]. Our results also found an association between CAD severity and serum troponin-I levels, suggesting its association with subclinical myocardial injury.

We recognize the limitations of the present study. The definition of high-risk CAD was based on published data linking severity and plaque characteristics. Although we had an adequate long-term monitoring with minimal losses, the number of patients studied was limited, as well as the incidence of some types of CV events, especially stroke. In addition, we did not conduct additional carotid US examinations during the follow-up or considered time-averaged levels of clinical and analytical parameters, thus our analyses relied on baseline measurements, limiting the validity of our results, considering the high variability of some serum parameters and the evolution of carotid atherosclerosis. The association between carotid atherosclerosis and calcified plaques with the incidence of cardiovascular events was lost in the multivariate analysis, which may be due to the low sample size and number of events. Thus, further studies with higher sample sizes and more events should confirm our findings. The evolution of patients that received a kidney transplant after the transplant was unknown since we discontinued the follow-up.

## Conclusions

In summary, we found that the echographic severity of CAD, and, especially, the presence of calcified plaques, are predictors of CV events, while IMT was not, in prevalent hemodialysis patients. These results challenges the general believe that calcified plaques are more stable, at least in this population. Larger prospective studies are needed to assess the relevance of CAD and, specifically, calcified CAD, as CV outcome predictors in ESRD patients. Carotid ultrasound is a simple and non-invasive tool that can help to evaluate the atherosclerotic burden in uremic patients and, perhaps, in combination with other imaging or biochemical markers may improve the CV risk prediction in this population. This is especially relevant in our hemodialysis patients in whom the Framingham risk score underestimates their actual cardiovascular risk [5].

## Supporting Information

S1 Fig Examples of CAD classification (A-D). A) IMT <0.9 mm. B) IMT >0.9 mm. C) Carotid plaque with stenosis <50%. D) Plaque with stenosis >50%.
(ZIP)

S1 Table Cardiovascular events and mortality during follow-up. (DOC)
