# Supplementary figures and images for: Carotid Atherosclerotic Disease Predicts Cardiovascular Events in Hemodialysis Patients: A Prospective Study
Source: PLoS One. 2015 Jun 1;10(6):e0127344. doi: 10.1371/journal.pone.0127344 (PMC4452075; doi:10.1371/journal.pone.0127344)

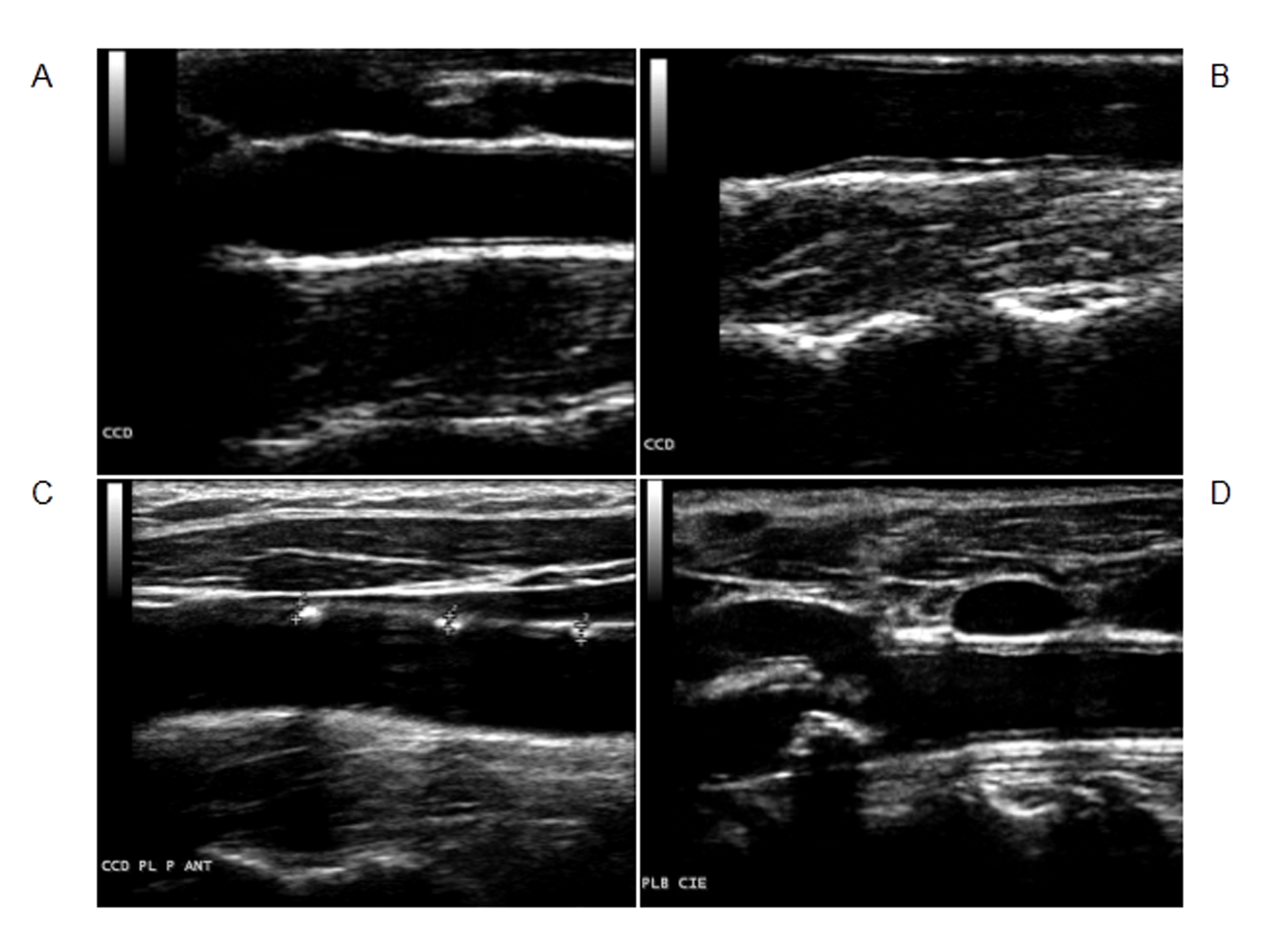

Supplement: S1 Fig — A) IMT <0.9 mm. B) IMT >0.9 mm. C) Carotid plaque with stenosis <50%. D) Plaque with stenosis >50%. (ZIP) [file pone.0127344.s001.zip › S2.Figure_S2(zip).tif]
