# Supplementary material for: Carotid Atherosclerotic Disease Predicts Cardiovascular Events in Hemodialysis Patients: A Prospective Study
Source: PLoS One. 2015 Jun 1;10(6):e0127344. doi: 10.1371/journal.pone.0127344 (PMC4452075; doi:10.1371/journal.pone.0127344)
Supplement: S1 Table — (DOC) [file pone.0127344.s002.doc]

**S1 Table.** Cardiovascular events and mortality during follow-up.

| **Cardiovascular events** | 32 (29.1%) |
| --- | --- |
| **Cardiac events** | 22 (69%) |
| Acute myocardial infarction | 7 (22%) |
| Angina | 2 (6%) |
| Arrhythmia | 6 (19%) |
| Heart failure | 5 (16%) |
| Sudden death | 2 (6%) |
| **Non-cardiac vascular disease** | 10 (31%) |
| Mesenteric ischemia | 1 (3%) |
| Peripheral vascular disease | 5 (16%) |
| Stroke | 2 (6%) |
| Transient Ischemic attack | 2 (6%) |
| **Overall Mortality** | 31 (28.2%) |
| **Cardiovascular deaths** | 12 (38.7%) |
| Acute myocardial infarction | 4 (13%) |
| Arrhythmia | 1 (3%) |
| Heart failure | 1 (3%) |
| Sudden death | 3 (10%) |
| Mesenteric ischemia | 2 (6%) |
| Peripheral vascular disease | 1 (3%) |
| **Non-cardiovascular deaths** | 19 (61.3%) |
| Infection | 8 (26%) |
| Neoplasia | 3 (10%) |
| Hemorrhage | 5 (16%) |
| Others | 3 (10%) |
